# Median effective dose (ED_50_) of esketamine combined with propofol for children to inhibit response of gastroscope insertion

**DOI:** 10.1186/s12871-023-02204-y

**Published:** 2023-07-18

**Authors:** Ming Su, Yichao Zhu, Shupeng Liu, Lijuan Song, Jiangtao Qu, Yong Zhang, Quanyi Zhang

**Affiliations:** 1 Binzhou Medical University Hospital, Binzhou, China; 2 Department of Anesthesiology, Binzhou Medical University Hospital, Huanghe-Str.2, Binzhou, China

**Keywords:** Esketamine, Propofol, Median effective dose, Gastroscopy, Sequential method

## Abstract

**Background:**

Propofol is the most commonly used drug for procedural sedation during gastroscopy. However, independent use of propofol can lead to increased dosage and additional side effects. Esketamine was found to be exceptional in combination with propofol for painless gastroscopy. No studies have calculated the median effective dose (ED_50_) of esketamine combined with propofol in pediatric painless gastroscopy. Here, we designed a research to study the ED_50_ of esketamine combined with propofol using the Dixon and Massey up-and-down sequential method for inhibiting the response of gastroscope insertion.

**Methods:**

Children who met the inclusion and exclusion criteria were included in this study. Propofol and esketamine were used as anesthetics for painless gastroscopy in children. To explore the ED_50_, the initial propofol dose was set at 3 mg/kg in all children. The first child was given an esketamine dose of 0.1 mg/kg, followed by 30 s of slow bolus injection propofol. If anesthesia induction failed (coughing or body movement of children during gastroscope insertion), the esketamine dose was elevated in the next child, with a interval difference of 0.05 mg/kg. Otherwise, if the anesthesia induction was successful, the next dosage was reduced by 0.05 mg/kg. The study was stopped if nine crossover inflection points were reached. The ED_50_ of esketamine was calculated using probit regression, and the blood pressure, pulse oxygen saturation, heart rate, recovery time, and side effects were recorded in all children.

**Results:**

A total of 26 children were included in this study. The ED_50_ of esketamine combined with 3 mg/kg propofol was 0.143 mg/kg (95% CI 0.047–0.398 mg/kg). The total consumption of propofol was 16.04 ± 5.37 mg. The recovery time was 16.38 ± 8.70 min. Adverse effects recorded were delayed awakening in two cases and increased oral secretions of another child during the examination inducing cough and hypoxemia (86% was the lowest).

**Discussion:**

The ED_50_ of esketamine was 0.143 mg/kg when combined with 3 mg/kg propofol for successful sedation in pediatric gastroscope insertion. This sub-anaesthetic dose of esketamine was safe and efficacious with few complications in pediatric painless gastroscopy.

**Trial registration:**

The study was registered at the Chinese Clinical Trial Registry (www.chictr.org.cn; registration number: ChiCTR2100052830 on 06/11/2021).

## Background

Unlike adults, children can be physically or psychologically traumatized during upper gastrointestinal endoscopy in an awake state. With the widespread development of comfort medicine, most pediatric gastroscopies are completed under sedation. Propofol is commonly used for procedural sedation for pediatric gastroscopy. However, the application of propofol alone for painless gastroscopy will enhance the dosage and side effects such as injection pain, respiratory depression, delayed recovery time, hypotension, and upper airway collapse [1]. In many cases, it is generally necessary to use propofol in combination with analgesic drugs to improve the anesthetic effect and reduce the adverse effects induced by a single drug.

Esketamine is the dextro-monomer of ketamine and the noncompetitive N-methyl-D-aspartate receptor antagonist. Esketamine has the advantages of rapid onset, analgesia, hypnosis, and mild respiratory depression. In recent years, esketamine was found to be exceptional in combination with propofol for procedural sedation during gastroscopy [2–4]. However, few studies have investigated the effective dose of esketamine for deep sedation in pediatric gastroscopy as an adjunct to propofol.

We designed a sequential enrollment study using the Dixon and Massey up-and-down method [5] to explore the median effective doses (ED_50_) of esketamine for painless gastroscopy in children with intravenous propofol injection.

## Methods

This study was approved by the Ethics Committee of the Affiliated Hospital of Binzhou Medical University, No. (2021KT-014). This project was registered in the Chinese Clinical Trial Registry on November 6, 2021, with the registration number ChiCTR2100052830.

This project was a sequential enrollment study. All pediatric patients were anesthetized and awakened in the Pediatric Endoscopy Center of our hospital. After the evaluation in the anesthesia clinic, the legal guardians of the children voluntarily signed the informed consent. Pediatric patients enrolled in this study aged1-14years, with a BMI of 18-25 kg/m^2^, American Society of Anesthesiologists (ASA) physical status I - II, and normal development. Exclusion criteria included: (1) allergy to esketamine or propofol; (2) definite difficult airway; (3) ongoing sedative therapy (e.g., propofol, morphine, fentanyl, sufentanil, midazolam, dexmedetomidine, ketamine) or recent use of sedatives (withdrawal time < 24 h); (4) cardiac or respiratory system diseases; (5) hepatic or renal malfunction; (6) developmental malformations.

### Anesthesia methods and gastroscopic procedure

All children were fasted for at least 6 h, and peripheral intravenous access was established. A total of 50 ml of Pronase solution (400 U/ml ) was drunk 5 min before endoscopy to eliminate foam and improve endoscopic graphic clarity, which was a proteolytic enzymes that can dissolve mucus in stomach. Oxygen was inhaled continuously at 3 L/min through a nasal catheter throughout the process. Children’s heart rate (HR), noninvasive blood pressure (right upper arm), pulse oximetry (SPO_2_), and electrocardiogram (ECG) were monitored during the gastroscopy and recovery. The patients were placed in the left lateral decubitus position with neck extension. Rescue medication and emergency airway equipment were prepared in advance. All clinical operations were performed by experienced endoscopists and anesthesiologists.

Esketamine (50 mg/2 ml, Jiangsu Hengrui Pharmaceutical Co., Ltd., China) was diluted to 50 ml by another researcher unaware of the research content. The diluted drug concentration was 1 mg/ml, which was easy to calculate and use. The first child was administered with 0.1 mg/kg esketamine; 30 s after injection, 3 mg/kg propofol (200 mg/20 ml, Beijing Fresenius Kabi Co., Ltd., China ) was injected slowly by an experienced anesthesiologist. After the child was sedated successfully, the pediatrician attempted to insert the gastroscope. The study was carried out with a sequential method. If the induction of anesthesia failed (significant body movement or choking) in the first child, the dosage of esketamine would increase to 0.15 mg/kg (0.05 mg/kg interval difference) for the next patient. Otherwise, the dosage of esketamine would decrease to 0.05 mg/kg. The study was not terminated until nine crossover inflection points were reached. According to the Modified Observer’s Assessment of Alertness/Sedation scale (MOAA/S) [6], all the children were sedated at the level of MOAA/S score = 1 (response only after trapezius squeeze stimulus). If sedation was unsatisfactory, 10–50 mg of propofol was intravenous injected until the endoscopy was completed. During the period of anesthesia maintenance, 0.5 mg/kg propofol was added according to the examination time and the patient’s response.

Once the child encountered bradycardia during the examination, 0.15 mg/kg atropine was immediately administered intravenously. If SPO_2_ ≤ 90%, mask-assisted ventilation was operated. If the child had hypotension (systolic blood pressure below 30% of baseline), 3–6 mg of ephedrine was administered. After the examination, the modified Aldrete scale was used to assess the departure of patients from the recovery room only if they reached a score of nine points or more [3].

### Observational indicators

The primary outcome was the median effective dose (ED_50_) of esketamine adjunct to 3 mg/kg propofol for pediatric patients to suppress the upper gastroscopy insertion response.

Secondary outcomes were as follows: total dosage of propofol; HR, SpO_2_, and blood pressure at time points before anesthesia induction (T_0_), immediately after esketamine injection (T_1_), immediately after propofol injection (T_2_), immediately after removal of gastroscope (T_3_), and 1 min after recovery (T_4_); endoscopy time; wake-up time; adverse reactions, e.g., nausea, delirium, delayed awakening, and respiratory depression.

The arousal time was defined as the period between gastroscopy extraction and the time when the children could open their eyes and cooperate. Arousal time exceeding 30 min implied delayed awakening. The Paediatric Anaesthesia Emergence Delirium(PAED) scale was used to evaluate the emergence delirium in children. SpO_2_ ≤ 90% indicated the presence of respiratory depression. Other unexpected complications were also recorded.

### Statistical analysis

The primary outcome ED_50_ and 95% confidence interval of esketamine were calculated using probit regression according to the data collected in this up-and-down sequential research. Due to the characteristics of this sequential study, the esketamine dose in the next patient depended on the success or failure of anesthesia induction in the previous patient. If the child showed significant choking, involuntary body movement, or airway obstruction preventing endoscope insertion, the anesthesia induction was recognized as “failure,” and the esketamine dose in the next child would be improved by 0.05 mg/kg. Conversely, the dosage was lessened by 0.05 mg/kg when it was “success.” Enrollment was stopped until the ninth crossover inflection point appeared.

Software SPSS version 26.0 (IBM Inc., Armonk, NY, USA) was used for statistical analyses. Normally distributed continuous data were expressed as the mean ± standard deviation. The Shapiro-Wilk test was used to determine whether the collected parameters were normally distributed. Non-normally distributed data were expressed as median (interquartile range, IQR). *P* < 0.05 was considered statistical significance.

## Results

A total of 26 children, including 14 boys and 12 girls, were included in this study. There were 18 cases of ASA class I and 8 cases of ASA class II, with an average age of 10.0 ± 2.17 years old[min 5, max 14] and a BMI of 19.46 ± 1.55 kg/m^2^[min 18.0, max 23.5]. The total consumption of propofol was 16.04 ± 5.37 mg. Hemodynamic indexes at T_0_-T_4_ are noted in Table 1.

**Table 1:** Changes in HR and MBP (right upper arm) at different time points: before anesthesia induction (T_0_), immediately after esketamine injection (T_1_), immediately after propofol injection (T_2_), immediately after gastroscope removal (T_3_), and 1 min after recovery (T_4_)

Hemodynamic Index	T_0_	T_1_	T_2_	T_3_	T_4_
Heart Rate (HR, min)	87.54 ± 17.03	87.77 ± 14_∙_63	97.23 ± 18.93	86.88 ± 15.23	85.62 ± 13.19
Mean Blood Pressure (MBP, mmHg)	71.38 ± 8.56	73.0 ± 10.18	67.90 ± 5.90	67.51 ± 7.11	72.26 ± 6.38

**Fig. 1:**
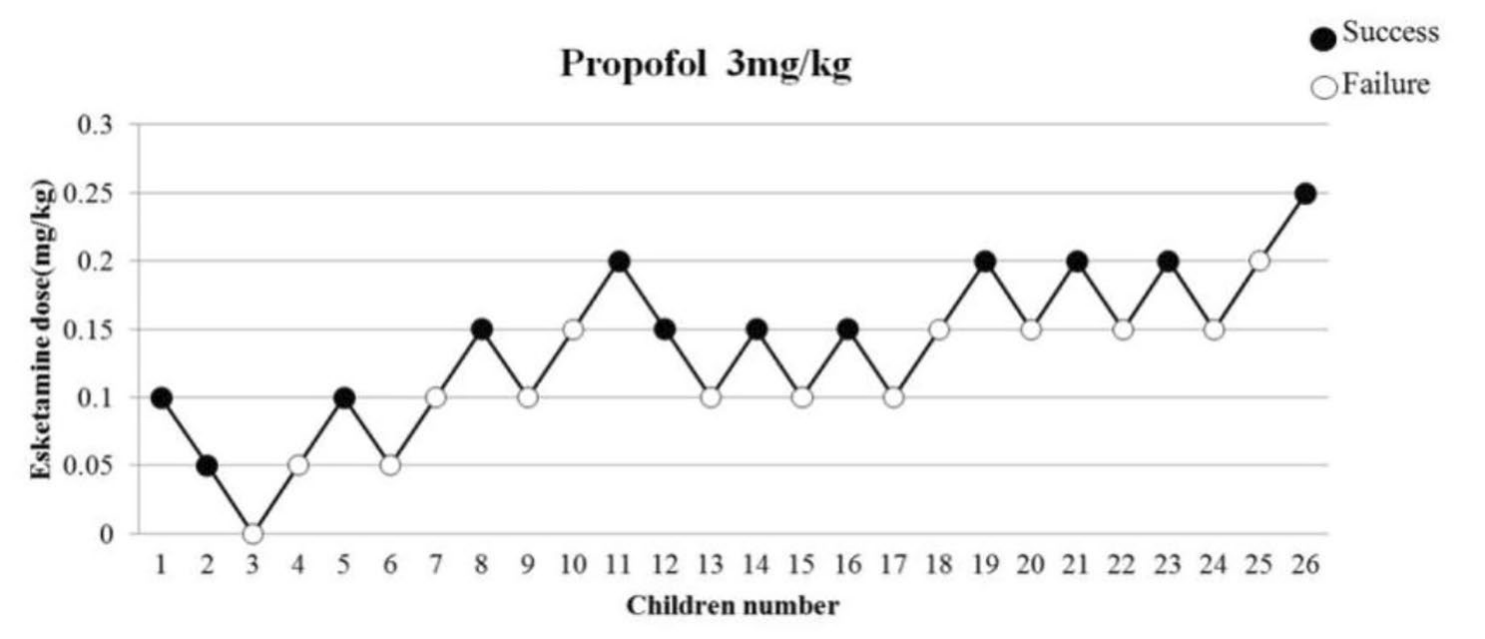
Dixon and Massey up-and-down plot line chart

**Fig. 2:**
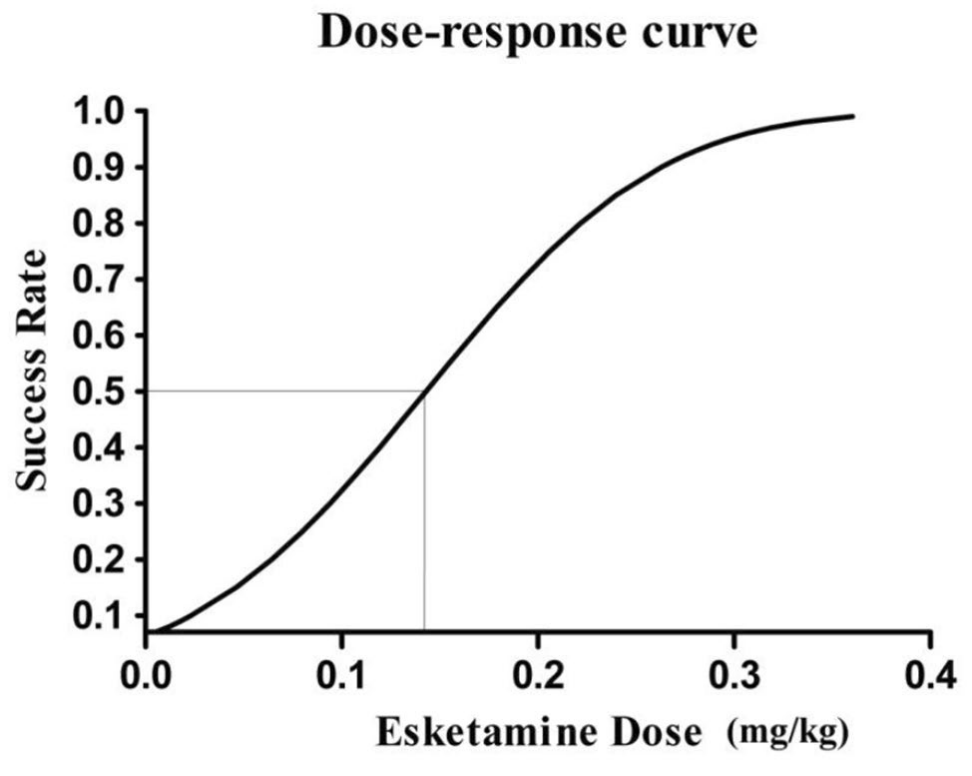
Dose-response curve of esketamine combined with 3 mg/kg propofol in this study for inhibiting the gastroscopy insertion response

At the beginning of our research, the first “failed” anesthesia induction appeared in the third case (0 mg/kg esketamine). The ninth turning point occurred in the 26th case, and subsequently, the experiment was terminated. Of the 26 children, 12 cases were “successful,” while 14 cases were “failed.” The ED_50_ of esketamine was 0.143 mg/kg (95% CI 0.047–0.398 mg/kg). The up-and-down series data are recorded in Fig. 1. The dose-response curve of esketamine in this study for inhibiting the gastroscopy insertion response is shown in Fig. 2.

Among the 26 children, the mean recovery time was 16.38 ± 8.70 min. Two patients delayed awakening at 32 min. No complication such as restlessness, nausea, or vomiting was found during the recovery period. One patient represented increased oral secretions during the examination, inducing cough and hypoxemia (86% was the lowest), which was relieved after oral suction and deepened anesthesia.

## Discussion

Esketamine, one of the two optical isomers of ketamine, is twice as effective as ketamine. The recommended dose of esketamine for intravenous anesthesia is 1.0-1.5 mg/kg [7], which, however, results in a marked increase in HR and blood pressure in children during clinical use, suggesting that esketamine is unsuitable for intravenous anesthesia in children alone. The combined use of different concentrations of esketamine (0, 0.25, 0.5, and 1 mg/kg) for gastroscopy in children greatly reduces the amount of used propofol, but 1 mg/kg esketamine will significantly intensify side effects [3]. Our study found that the 3 mg/kg propofol combined with low-dose esketamine could effectively avoid the occurrence of related side effects. The adverse reactions were found as follows: 2 cases of delayed awakening (32 min) and 1 case of hypoxemia during the operation (minimum 86%) due to the irritation of the throat by secretions. The results confirmed the safety and feasibility of 3 mg/kg propofol combined with low-dose esketamine for gastroscopic sedation in children.

Several regimens are available for sedation in pediatric gastroscopy, among which the most widely used is propofol, which features a rapid onset, efficacy, and recovery time [8]. A study involving 36,516 children with procedural sedations indicated that propofol provided deep sedation but brought about more complications in children undergoing gastroscopy [9]. Propofol alone is difficult to produce satisfactory sedation during gastroscopy. Anesthesiologists frequently use propofol for painless endoscopy in combination with opioids, benzodiazepines, dexmedetomidine, and esketamine [10–12]. The combination of opioids and propofol could provide strong and effective analgesia but also aggravate the onset of respiratory depression, especially in children [13]. Small-dose esketamine combined with propofol was expected to improve cardiorespiratory stability for painless gastroscopy [4]. Esketamine has been identified to stimulate the respiratory center and counteract respiratory depression to some extent caused by the rapid propofol injection [14, 15].

Some studies have explored the dose of propofol in combination with different doses of esketamine used in pediatric painless gastroscopy. Zheng XS [3] and J. Hayes [16] evaluated four doses of esketamine adjunct to propofol. Abandoning their primary target ED_50_ of propofol, they found that 1 mg/kg esketamine enhanced the incidence of nausea and visual disturbances. In our study, esketamine dose was explored in the range of 0-0.25 mg/kg, and none of the above adverse reactions were found in all children during follow-up. So far, the optimal esketamine dose in conjunction with propofol for pediatric gastroscopy has not been identified. The starting dose of propofol in the study of Zheng XS and his colleagues was 2.5 mg/kg in the 0.25 mg/kg esketamine group. Based on our pre-experimental explorations, we designed 3 mg/kg as the starting dose of propofol, which was also utilized in our daily work experience. In this study, we did not find propofol-induced respiratory depression. In addition, we discovered an unexpected result that when the esketamine dose was greater than 0.15 mg/kg, the injection pain of propofol was significantly relieved.

In our study, the main adverse reactions were delayed awakening and hypoxemia induced by increased secretion during the examination. Except for two cases of delayed awakening, all pediatric patients were awakened within 30 min. Elevated secretions could be relieved with anticholinergic drugs to avoid the occurrence of cough and hypoxia. Hypotension is the main propofol-induced cardiovascular complication [17] but was not obvious in our research, possibly because the sympathomimetic effect of esketamine counteracted the hypotensive effect of propofol [18]. The most common reaction in the 14 “failure” cases of the greatest value was involuntary body movements, which could be ameliorated by the addition of propofol. An involuntary body movement response of the child patient after induction often indicated the induction failure, and thus additional propofol was required to perform the endoscopic insertion.

The first noted limitation was that we only calculated the ED_50_ of esketamine using the up-and-down method but did not explore the ED_95_ and its 95% CI. Pediatric patients are prone to perioperative hypoxia events because of their unique airway anatomy. In addition, the endoscope insertion can irritate the throat, thereby enhancing the incidence of hypoxia. Calculation of ED_95_ might involve the application of large doses of esketamine, which has been proved to cause significant side effects. A wide 95% confidence level can result in ethically impermissible clinical accidents. Considering these risk factors, we did not explore the ED_95_ dose in a direct manner [19]. The present study was single-center and small-sample sequential and thus did not include multiple confounding factors. We did not explore overweight children and these developmental abnormalities. With the rising incidence of obesity in children, further research should focus on the optimal dose of the combination of esketamine and propofol in this special population. In our study, the children enrolled did not contain the low age group (aged below 4), which also needs more research.

## Conclusion

In conclusion, a low dose of esketamine (ED_50_ 0.143 mg/kg (95% CI 0.047–0.398 mg/kg)) combined with propofol (3 mg/kg) can provide a satisfactory sedative efficacy with excellent safety and feasibility in pediatric painless upper gastroscopy. More research remains to be conducted when combining esketamine and propofol under different confounding conditions.

## Data Availability

The data of this study are available from the corresponding author by reasonable request.

## Abbreviations

ED_50_: Median effective dose
SPO_2_: Oxygen saturation
ASA: American Society of Anesthesiologists
